# Sleeping but struggling: a qualitative study of the lived experiences of sleep in student-athletes

**DOI:** 10.1038/s41598-026-55657-9

**Published:** 2026-06-02

**Authors:** Sandy M. B. Wilson, Luciana De Martin Silva, Martin I. Jones, Stephen B. Draper, John K. Parker

**Affiliations:** 1 College of Medical, Veterinary & Life Sciences, University of Glasgow, Glasgow, G12 8QQ UK; 2 Department of Sport, Hartpury University, Gloucestershire, GL19 3BE UK

**Keywords:** Health care, Psychology, Psychology

## Abstract

A growing body of evidence indicates that student-athletes experience suboptimal sleep health across multiple dimensions. However, limited research has explored sleep from the student-athlete’s own perspective. This study examined the lived experience of sleep health in a cohort of student-athletes known to demonstrate suboptimal sleep outcomes. Semi-structured interviews were conducted with 12 British rugby union student-athletes. During interviews, participants were presented with personalised sleep data from a prior actigraphy assessment, which served as a visual elicitation tool to facilitate reflection and discussion. Interview transcripts were analysed using reflexive thematic analysis. Nine lower-order themes were identified and organised into three higher-order themes: *irregular patterns*, reflecting structural and social demands that constrained control over consistent sleep; *managing sleep behaviours*, encompassing individual behaviours that often conflicted with established sleep recommendations; and *connection to health*, describing bidirectional relationships between sleep, wellbeing, and performance, whereby inadequate sleep contributed to negative physical, cognitive, and emotional outcomes. Overall, student-athletes identified multiple upstream influences that adversely affected their sleep health, with wide-ranging consequences for wellbeing. These findings contextualise previous quantitative evidence of poor sleep in student‑athletes and may inform the subsequent development of targeted sleep interventions grounded in behaviour change theory.

**Supplementary Information:**

The online version contains supplementary material available at 10.1038/s41598-026-55657-9.

## Introduction

Sleep health has emerged as an important concept over the last decade that emphasises sleep as a public health matter relevant to all individuals^1^. This viewpoint posits that sleep health exists on a continuum and is multidimensional, whereby improvements in one or more dimensions can yield downstream benefits for health and well-being^2^. Framing sleep in this manner is particularly relevant for populations where sleep may not present as a disorder but is still characterised by poor sleep outcomes underpinned by various upstream factors^3^. One such population are student-athletes – also commonly referred to as dual-career athletes – that combine university-level education with performance sport. Student-athletes often present with suboptimal sleep health across several dimensions such as short sleep durations and irregular sleep patterns as a product of these dual demands^4^. Poor sleep can result in a range of undesired downstream consequences for student-athletes related to sport performance, academic attainment, injury risk, and wellbeing^4^.

Questionnaires are typically used as the primary method to identify factors influencing sleep health in research, such as the Athlete Sleep Behaviour Questionnaire^5^. However, while these approaches can identify maladaptive sleep behaviours, they provide limited contextual insight into why such behaviours occur. Understanding these underlying influences may benefit from qualitative approaches that enable a more in-depth exploration of participant perceptions and experiences^6^. Despite this, qualitative research exploring sleep health remains sparse across populations. For instance, in systematic review mapping evidence on sleep in students within higher education, only 2% of studies employed an exclusively qualitative design^7^. Interviews also provide an opportunity to incorporate tools that may enhance reflection and discussion, such as presenting individual sleep data as a form of visual elicitation. While this approach has previously been used as a feedback mechanism in intervention studies^8^, its use as a method for eliciting participant experiences of sleep health remains largely unexplored. Consequently, there is a need to understand the lived experience of sleep in student-athletes to identify modifiable upstream factors influencing sleep health and inform the development of targeted interventions.

Qualitative studies that have been conducted on student populations have highlighted numerous causes of suboptimal sleep health including late night studying, part time work, accommodation-related noise, and social life, with downstream consequences of poor sleep health included poorer mental health and wellbeing and academic difficulties^9–12^. Similarly, calls for greater use of qualitative and mixed method approaches within performance athletes appear to have had limited uptake^13^, but such studies have highlighted barriers related to stress, injury, training, and sleep environments^14–16^. While none of these barriers are inherently surprising based on research adopting quantitative approaches, they can add understanding as to *why* these occur. For instance, Foulkes et al.^9^ found that while students knew that late-night socialising would negatively impact sleep, they were willing to compromise on sleep for the perceived social benefits of doing so. While there have been a few qualitative studies that have considered health in student-athletes where sleep has been raised by participants as one of many health behaviours^17–20^, there appears to be no research that has considered sleep health as the primary topic. Therefore, this study aimed to use qualitative methods which incorporate visual elicitation to explore the lived experience of sleep health in a cohort of student-athletes.

## Methods

### Design

This study employed an exploratory qualitative design to examine the lived experience of sleep health in student-athletes using semi-structured interviews incorporating visual elicitation. The study was informed by a constructivist epistemology and relativist ontology, recognising that experiences of sleep health are subjective, context-dependent, and shaped through social, behavioural, and environmental influences. This philosophical position was considered appropriate because the study aimed to explore how student-athletes interpreted and attributed meaning to their sleep experiences, rather than identify a singular objective reality.

Reflexive thematic analysis (RTA) was selected as the analytical approach because it aligns with constructivist assumptions and acknowledges the active interpretative role of the researcher in theme development^27,28^. RTA was considered particularly suitable given the exploratory nature of the study and the aim of identifying patterns of shared meaning across participant experiences while retaining flexibility in interpretation. The methodology and reporting were informed by the Consolidated Criteria for Reporting Qualitative Research (COREQ) guidelines^21^.

### Participants

Participants were recruited from a previous research study in which sleep patterns were assessed over a fortnight using wrist-worn actigraphy^22^. This sampling approach was used because participants demonstrated suboptimal sleep characteristics in the preceding study and had existing actigraphy data available for use within the interviews. Participants were approached through Microsoft Teams using convenience sampling, with 12 completing interviews. Data saturation was not used to determine sample size, as reflexive thematic analysis does not assume a finite set of discoverable themes but instead involves an iterative and interpretative process in which themes are actively generated^23^. The research team considered the twelve interviews sufficient to generate meaningful and nuanced patterns of shared meaning relevant to the study aims, while also remaining consistent with recommendations from previous qualitative interview research^24^. On this basis, no further participants were recruited from the eligible sample.

No participants declined to participate or withdrew. All participants were from the same team participating in British University and College Sports Super Rugby, the highest level of student-athlete competition in the United Kingdom. Participant ages ranged from 18 to 25, and ethnicity was self-reported as White (*n* = 10) and Black, Black British, Caribbean, or African (*n* = 2). The study received institutional ethical approval from Hartpury University Ethics Committee (ETHICS2023-01) and all student-athletes provided written consent prior to participation.

### Interviews

Individual semi-structured interviews were conducted either in-person (*n* = 10) or online (*n* = 2), with all interviews recorded using Microsoft Teams and audio recordings lasting 24–52 min (median = 40 min). Interviews were conducted by the lead researcher [SMBW], who at the time was a PhD researcher and part of the squad’s multidisciplinary sport staff at the time of data collection, and participants were familiar with the interviewer and the research purpose. Consequently, reflexive consideration was given to how prior relationships, assumptions, and contextual knowledge may have influenced data collection and interpretation. While familiarity with participants facilitated rapport and openness during interviews, there was also potential for socially desirable responding and researcher assumptions shaping follow-up questioning and interpretation. To minimise these influences, participants were reminded that responses would remain confidential, that there were no right or wrong answers, and that honest reflections were encouraged irrespective of perceived expectations.

An interview guide was designed to enable participants to direct the conversation while maintaining consistency across interviews^25^. The guide was refined following two pilot interviews with other student-athletes to check understanding and improve clarity. The guide was split into three sections, with section one and two designed to elicit information on sleep health, and section three designed to explore previously raised topics in greater depth. The full guide is available as Supplementary Material.

**Fig. 1:**
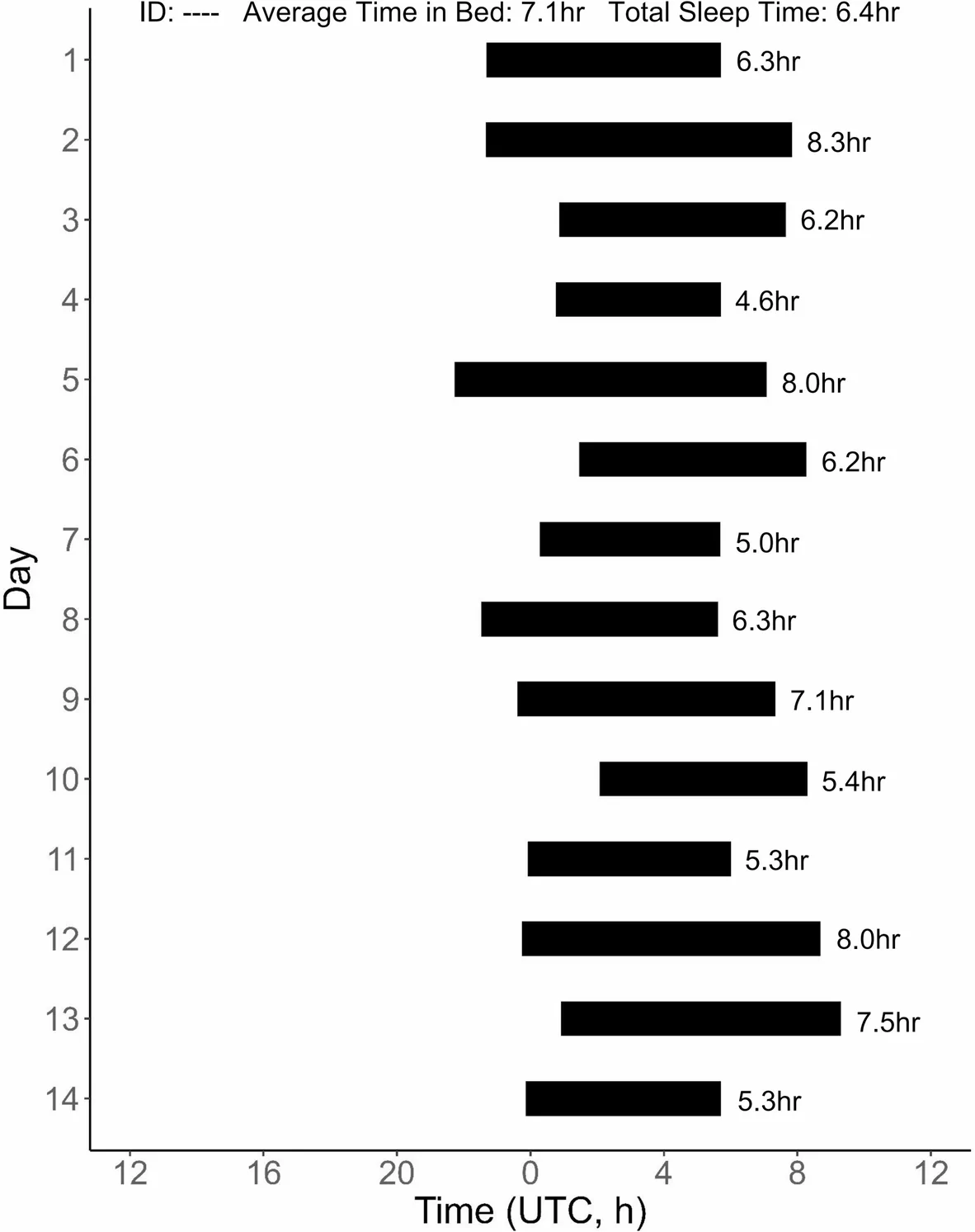
Example of visual sleep feedback provided to participants. Each bar represents the sleep episode and the duration from sleep onset time to wake up time.

In section one, participants discussed their sleep in relation to being an student-athlete (e.g., the associated challenges and comparisons with non-athlete peers) and reflected on their satisfaction with their sleep practices. The interviewer noted any relevant topics to revisit in greater depth. In section two, participants were given a printed handout presenting their own sleep patterns from previous actigraphy study^22^ (Fig. 1). This handout served to act as a visual elicitation tool to prompt reflection and discussion on the experience of each participant. Recorded sleep patterns were taken between one and three weeks before the interview was conducted and was created using an adapted output from the GGIR analysis package^26^, and feedback had not been seen by the participants before this point. The interviewer explained the handout format, including how to identify sleep onset, offset, and total sleep time. No additional feedback was provided at this stage. Participants were asked to reflect on the data, share initial thoughts, and provide context for specific days (e.g., factors underpinning short or long total sleep durations). As in section one, topics deemed relevant for further discussion were noted.

In section three, previously noted topics were explored further. Participants elaborated on these topics and provided practical examples, discussing whether they perceived them as facilitators or barriers to sleep health. For barriers, participants were asked what changes could address them and improve sleep. Any novel topics raised were also explored in the same manner. Before concluding, participants were invited to raise any further points they deemed important. After recording ended, the interviewer provided approximately 10 min of individualised feedback on sleep data and offered practical advice.

### Data analysis and rigour

Audio recordings were transcribed using a two-step process: initial transcription was automated through Microsoft Teams, followed by manual verification to ensure accuracy. Each recording was carefully listened to, and the original transcript was edited as needed. Transcripts were then uploaded to NVivo 14 (Lumivero, Denver, CO) for analysis. Participants had the option to review transcripts for accuracy; none opted to do so. Interview transcripts were de‑identified prior to analysis with the removal or modification of names, institutional identifiers, team‑specific details, and any contextual information that could reasonably lead to participant identification.

Interview transcripts were analysed using the six-step process of reflexive thematic analysis described by Braun and Clarke^27^. In Step 1, transcripts were listened to and read twice to immerse the researcher in the data and enhance understanding. In Step 2, initial inductive, semantic codes were generated from each transcript, followed by a second round of coding to refine, expand, and collapse data. In Step 3, codes were grouped into potential themes based on patterns and shared meanings. Step 4 involved reviewing these themes to ensure they addressed the research question. Steps 3 and 4 were revisited iteratively until the lead researcher was confident the themes accurately reflected the interview content. Clear clusters of higher-level themes were identified, with earlier themes re-categorised as lower-level themes under these overarching themes. Step 5 involved naming and defining the higher-level themes, followed by the final write-up in Step 6.

To support reflexivity and enhance the trustworthiness of the study, the lead researcher [SMBW] maintained personal reflective notes throughout data collection and analysis, documenting interview dynamics, assumptions, and evolving interpretations. Given the researcher’s existing role, ongoing reflexive consideration was given to how prior relationships, contextual familiarity, and researcher assumptions may have shaped both participant responses and data interpretation. Discussions with fellow researchers acting as critical friends were used to challenge assumptions, explore alternative interpretations, and encourage reflexive engagement throughout theme development^29^. These discussions focused on the coherence of interpretations with the dataset and the transparency of analytic decision-making rather than seeking consensus or inter-rater agreement.

## Results

**Fig. 2:**
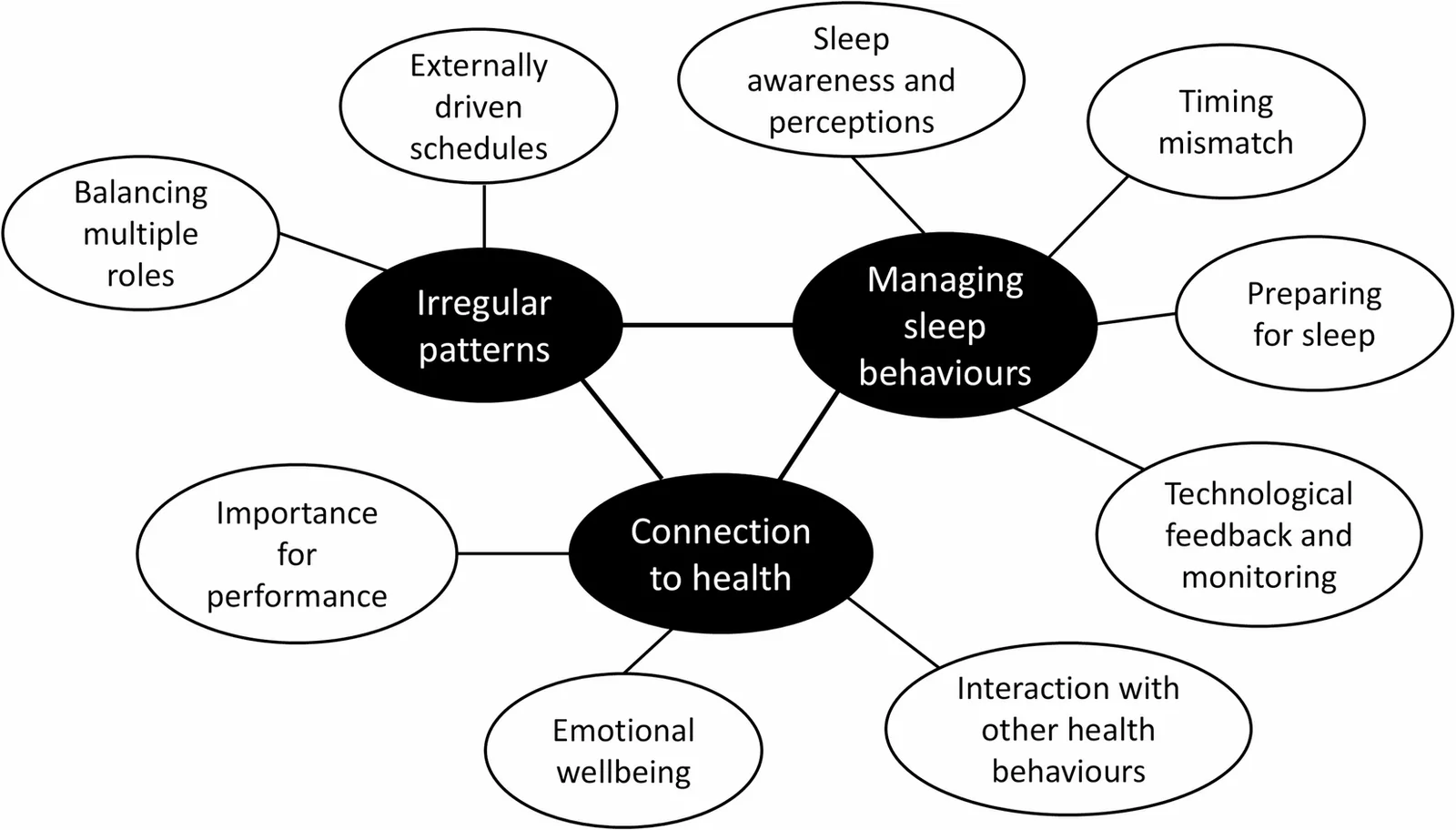
Thematic map of higher-order and lower-order themes.

Three higher‑order themes were developed from the analysis, with nine lower-order themes. Together, these describe how student‑athletes perceived their sleep as shaped by externally imposed demands, individual sleep‑related behaviours, and downstream consequences for health, wellbeing, and performance. While these themes are presented separately, participants’ accounts frequently reflected their interconnected nature (Fig. 2).

### Theme 1: Irregular patterns

The theme of *irregular patterns* reflects aspects of being a student-athlete that created a perceived inability to maintain regular sleep timing and duration. These patterns were largely attributed to social‑ and structural‑level demands over which participants felt they had limited control. As such, irregular sleep was often seen as ‘part of the package’ of being a student‑athlete, despite being a source of frustration and fatigue.

#### Theme 1a: Externally driven schedules

All participants highlighted early morning training sessions, typically beginning at around 06:30, as a major determinant of their sleep patterns. Training was often scheduled before academic commitments and occurred on irregular days across the week, leading to fluctuating sleep and wake times. While some participants suggested they had adapted to early starts – “Training in the morning is hard, like getting up, but you kind of get used to it” (Participant H) – all reported tiredness on at least some occasions following training:

So obviously for some boys it can be a bit of a challenge around maybe feeling quite tired a lot the day [after training]. Like that’s maybe one thing that I do feel is like in the day I do get a bit tired. So, I do occasionally nap, or maybe some things I am less, less inclined to do (Participant E).

Daytime napping was commonly reported in response to accumulated sleep loss. These naps were often described as unplanned and, at times, disruptive to academic engagement. One participant noted “[I would] look at my phone, see I have a lecture at like one and be like nope, just put it back down and go back to sleep” (Participant B). In contrast, participants described more regular sleep patterns during holiday periods or phases of the season with later training start times, during which the need for daytime napping was reduced.

#### Theme 1b: Balancing multiple roles

In addition to training schedules, participants described how managing multiple roles further constrained sleep opportunity. Early training followed by academic commitments often resulted in extended days, “until about 18:00, twelve hours later, at least” (Participant H), limiting opportunities for recovery during the week and contributing to accumulated sleep debt that was typically compensated for at weekends. For eight participants, part‑time employment added an additional layer of demand, further reducing time available for sleep: “With a social life – like you play rugby, do uni[versity], and I am working weekends. So yeah, I don’t really take a day off and sleep the next day to catch up” (Participant J).

Participants also highlighted team‑based socialising as an important element of the student‑athlete role. While generally not viewed as disruptive, mid‑week social events following matches—particularly on Wednesdays—were consistently identified as an exception. These occasions often involved late bedtimes and alcohol use, resulting in shortened sleep and lingering fatigue:

If I go out on the Wednesday night I find, if I’m being honest, it’s probably going to bed about 04:00 or 05:00 in the morning if l stay out. Like, if I’m really shattered then I’d probably be 01:00 to 02:00-ish. But the whole Thursday is like, even if it’s not hungover I’m just fatigued and we’ll have Friday. OK, I cope with it, but I think my main issue from like that lack of sleep (Participant G).

### Theme 2: Managing sleep behaviours

The theme of *managing sleep behaviours* relates to various reported sleep-related behaviors that can either support or hinder the ability to implement recommended sleep practices. Unlike irregular patterns, participants felt that they have greater control over these factors and are aware of their impact on sleep health. However, they would often engage in behaviors that counter typical sleep recommendations.

#### Theme 2a: Timing mismatch

Five participants described setting specific sleep targets before training days, either in terms of total sleep duration — “I will always look for around seven hours” (Participant A) — or a set bedtime. However, attempts to go to bed earlier frequently resulted in difficulty initiating sleep. This was attributed to conflicting sleep preferences, with many participants reporting that they naturally felt more alert later in the evening: “I don’t really feel tired when you go to bed. So, I’ll just be like scrolling on my phone, trying to get tired to be able to fall asleep” (Participant C).

This mismatch between required and preferred sleep timing contributed to prolonged sleep onset on nights preceding early training sessions, with one participant stating, “I’ll turn my phone off… half an hour later, I’m still like wide awake and I’m just not dropping off” (Participant G).

#### Theme 2b: Preparing for sleep

Phone use in bed was raised in all interviews and commonly described as part of participants’ pre‑sleep routines, despite awareness that it conflicted with general sleep hygiene guidance. Experiences of its impact varied. Some participants felt phone use interfered with sleep initiation, whereas others perceived it as beneficial, helping them relax — “almost wind down and let me go to sleep” (Participant H).

Other reported sleep preparation practices included limiting light exposure, using breathing techniques, and enabling sleep modes on electronic devices. These behaviours appeared informal and inconsistent in a similar manner to schedules where preparation for sleep was more consistent on nights before training but not followed through on nights preceding free days.

#### Theme 2c: Technological feedback and monitoring

Four participants reported monitoring sleep on some occasions using smartwatches or applications, though this was generally infrequent and motivated by curiosity rather than purposeful behaviour change. None described systematic sleep tracking or formal monitoring by the team. Instead, sleep adequacy was assessed subjectively, based on how participants felt during the day. One participant stated “I feel like I won’t go on to the app then like look and see because I’ve had this amount of sleep. I need a nap. My body will just naturally tell me that*”* (Participant I).

#### Theme 2d: Sleep awareness and perceptions

Perceptions of sleep knowledge varied across participants. While some background knowledge was reported by most student-athletes, only one reported acquiring this through academic study. It was felt sleep was rarely discussed in meaningful ways within their sporting environment, with one student-athlete stating, “I can’t say I know a lot about sleep, and I can’t say we speak about sleep enough” (Participant I). Sleep was on occasion described as an implicit or avoided topic. Although coaches were not perceived as dismissing its importance, participants suggested that limited emphasis was placed on sleep due to the perceived gap between ideal practices and what was realistically achievable within their schedules.

### Theme 3: Connection to health

The theme of *connection to health* reflects perceived relationships between sleep, physical health, psychological wellbeing, and daily functioning. These relationships were often described as bidirectional, with poor sleep contributing to outcomes that further disrupted sleep and preventing student-athletes from excelling in both academic and athletic roles.

#### Theme 3a: Interaction with other health behaviours

When discussing the downstream effects of poor sleep, eight participants raised the interaction with diet. This relationship was described in both directions: eating late, often due to scheduling constraints, made it harder to fall asleep – “One night I might have ate too much as well, so I felt a bit sick, I couldn’t go to sleep” (Participant L) – while fatigue from inadequate sleep led to poorer dietary choices:

I’m not picking bad foods, sometimes, it’s just quick food. So… I’ll pick up a sandwich from the shop or something like that. But yeah, that’s still costing me as well. So like, I’m probably sacrificing myself in cost and nutrition (Participant F).

This created a vicious cycle, where poor sleep affected health-related choices, which then impacted sleep the following night. A couple of participants also highlighted links between sleep and physical activity, noting that poor sleep made training feel more demanding, leaving them “less energised, and it’s more of a struggle” (Participant D).

#### Theme 3b: Importance for performance

The importance of sleep for athletic performance was universally acknowledged. Rather than being specifically related to performance outcomes, sleep was framed primarily as a recovery tool, essential for muscle repair, growth, and sustaining energy and alertness. Four participants discussed academic performance explicitly, in which a similar beneficial effect of good sleep was reported. It was perceived that academic stress disrupted sleep, and in turn poor sleep made it harder to concentrate during lectures:

Lectures like, I am trying to concentrate on what he’s trying to say, and I’ve never had it ever before. So, like my heads, my heads tilting forwards and I’m like, ‘oh, my God, I can’t stay awake.’ Like I’m rubbing my eyes trying to like snap out of this sort of thing (Participant F).

#### Theme 3c: Emotional wellbeing

Ten participants also identified links between sleep and mood, with one other reporting no clear link. Short nocturnal sleep, often due to early training or peer‑related disturbances, was associated with irritability, frustration, and grogginess. These mood changes were attributed directly to sleep loss and were resolved following the opportunity to obtain sufficient sleep. Napping was commonly described as a means of emotional recovery:

They’ve woken me up at like 5:00 o’clock in the morning and whatever shenanigans, gone back to bed and then like, they’re up again at like 9:30, I’m stroppy the entire rest of the Sunday. And then even on, in a training session, if I’ve not had good night’s sleep, I’m finding myself very, very snappy with people. And then it just ruins it, the same for the rest of the day… but the moment I’ve got nothing to do I’ll have a nap and then I find having a nap does help me like, sort my mood out (Participant G).

## Discussion

This study aimed to explore the lived experience of sleep in a cohort of student-athletes that are known to present with suboptimal sleep health from a previous study by using qualitative methods which incorporate visual elicitation. The themes identified following the RTA identified three themes – irregular patterns, sleep hygiene practices, and holistic health – and nine sub-themes related to the lived experience of sleep health in student-athletes, many of which could serve as viable targets for intervention design. Furthermore, the study illustrated how semi-structured interviews incorporating visual elicitation based on personal sleep data can elicit rich insights into sleep health that can complement quantitative approaches.

The identification of irregular patterns as a higher-order theme was unsurprising, given that the main finding from the previous study was that sleep in this cohort were highly irregular as a consequence of training in the early morning^22^. However, participants emphasised that the issue is not just morning training, but the constant switching between early starts and preferred wake times, creating inconsistent sleep patterns. Although the extent of these difficulties could be unique to this cohort, morning scheduling is common in student-athletes to accommodate academic timetables, positioning it as a key modifiable factor to improve sleep consistency. The perceived workload of balancing sports and academics is often cited as restricting sleep in American student-athletes^30^, although participants highlighted further factors that may contribute towards restricted sleep opportunity. This included working part-time jobs to support their studies that are often placed at night (e.g., bar work), as supported by Barone^12^ who showed a similar difficulty in balancing work, university and sleep in students. Additionally, participants highlighted the role of alcohol consumption as part of the social structure in university rugby, as previously identified in research^31^. While this was perceived as important for team cohesion, in a similar manner to interviews with students by Foulkes et al.^9^ where the social benefits outweighed the negative impact on sleep health, such events could disrupt sleep patterns for multiple nights.

The sleep hygiene practices theme encompassed various behavioural and environmental strategies to promote sleep. Many participants reported setting target bedtimes to ensure adequate sleep opportunity but struggled to maintain these. This likely reflects a mismatch between required early bedtimes and the circadian phase delay commonly seen in young adults, whereby biological sleep preference shifts toward later sleep and wake times^32^. Participants’ lack of awareness of the circadian contribution to sleep regulation may contribute to this issue. Research indicates that athletes often lack the knowledge needed to apply sleep hygiene effectively^5^, and education-based interventions have been shown to improve sleep^33^. Pre-bed routines varied in the current study, with many following common sleep hygiene guidelines such as reducing light exposure. Participants frequently cited the use of electronics in bed, which is a common behaviour found in junior athletes^14^. While phone use was perceived by participants as a negative behaviour, evidence is equivocal on the extent to which typical device use impacts sleep^34^. Despite high technological literacy, few participants used devices to monitor sleep, even if they wore sleep trackers. Although such devices have accuracy limitations^35^, the feedback from wearables can provide longitudinal data that can be used to understand current practices and promote healthier sleep behaviours. Instead, participants relied on intuition and how they felt during the day to assess their sleep.

The holistic health theme reflected how sleep affects various downstream aspects of health and wellbeing, which, in turn, influence future sleep behaviour. One key issue raised was the relationship between sleep and nutrition. For example, participants noted poorer nutritional decisions following sleep loss, which has been documented in research^36^. The relationship between sleep and athletic performance was also expected, given the participants’ competitive level. While evidence on sleep and performance is mixed, cognitive performance is more sensitive to sleep loss than physiological performance^37^. Similarly, participants identified a link between sleep and academic performance, in line with research showing that shorter sleep durations are associated with poorer academic outcomes in student-athletes^38^. Across interviews, there was also a sense of regret that participants could not obtain enough sleep to facilitate their performance, which aligns with previous research by Robbins et al.^39^, where insufficient sleep was retrospectively considered a common health regret at the end of a season. The emotional impact of sleep was another notable issue, with participants reporting more intense negative emotions, such as anger, after poor sleep. However, these emotions did not appear to affect sleep the following night, which aligns with findings that daily fluctuations in sleep quality impact emotional states the next day, but not vice versa^40^.

A benefit of the methodological approach employed in this study was the presentation of sleep data as a form of visual elicitation within interviews to prompt discussion. A limitation of traditional qualitative data collection strategies is the communication of subjective health experiences, and whether an interview alone produces data of the desired quality and depth^41^. This consideration could be particularly pertinent in sleep, where the altered state of consciousness may make it more difficult for participants to retrospectively reflect upon and recall sleep compared to other health behaviours during wake. The visual elicitation implemented in the interviews served to increase the trustworthiness of the study by using multiple methods of data collection and enabled participants to move beyond verbal modes of thinking and generate deeper personal insights into sleep health^41^. Our experience during this study was that whereas the discussion in section one (before visual elicitation) was broader in scope and generalised to the general athlete population, the introduction of the visual feedback in section two prompted discussion that was more nuanced and richer in depth. The visualisation enabled comparison between days and facilitated discussion on the factors underpinning any differences, and response were more individualised to their personal experience of sleep. This aligns with previous research by Griggs et al.^42^ where sleep reports generated from actigraphy were used to guide the generation of sleep health goals in young adults with diabetes and found the visualisation to be a useful strategy to elicit an effective goal setting strategy. While the use of actigraphy to generate personalised sleep reports is a time-intensive method, there are alternative methods that could be used to create visual elicitation reports. For example, some sleep diaries such as adopt a visual approach where sleep and related behaviours (e.g., exercise) can be marked across the 24-hour day by the participant and used within subsequent interviews^43^.

A further benefit of the approach taken in this study is the ability to use the findings to conduct a behavioural diagnosis and design targeted interventions that utilise behaviour change theory and are driven directly by participants. Conducting a behavioural diagnosis addresses a common criticism in health intervention research, where interventions are often designed based on assumptions of expected behaviours rather than empirical examination^44^, or what has been termed the ‘ISLAGIATT’ principle (‘It seemed like a good idea at the time’). The identified lower-order themes presented in this study were used to design a behavioural intervention through mapping onto the COM-B model of behaviour change to identify the behavioural components that were perceived as barriers, then designing a targeted intervention using the Behaviour Change Wheel^45,46^. This approach to intervention design is commonplace in other health behaviours, although there is limited evidence to suggest widespread implementation of COM-B or other behaviour change theory when designing sleep health interventions^47^. The process of intervention development and pilot testing are available as a separate manuscript which showed preliminary evidence to support the effectiveness of this approach, but also the need to design interventions that target al.l identified behavioural barriers^48^.

There are some points that should be considered when interpreting these findings. Participants were recruited through convenience sampling from those who completed the previous actigraphy-based study^22^. While the number of respondents was deemed sufficient for the RTA to capture the lived experiences of student-athletes, it is feasible that the experiences of participants are not reflective of the entire squad. For instance, individuals with poorer sleep may have been more motivated to seek feedback and advice compared to those with no sleep issues. In addition, while the themes identified are likely to be shared across many student-athlete cohorts, there may be influences that were specific to this group of participants, such as the sport played or the institution studied at. This reinforces the importance of utilising a similar methodological approach where themes are to be used to create targeted interventions, rather than assuming that the same behavioural barriers exist. Furthermore, while visual elicitation appeared to support participant reflection during interviews, the specific influence of personalised sleep visualisations on the depth or nature of participant responses was not formally evaluated.

## Conclusion

This study offers insight into the lived experience of sleep health in student-athletes and helps contextualise previous research that has shown student-athletes to have suboptimal sleep when assessed using quantitative measures. Participants described how their sleep is shaped by structural demands, individual behaviours, and broader health and performance considerations. However, it was evident that these factors interact to result in poorer sleep than participants desired during the competitive season when combining academic study and sport performance. The findings highlight the value of qualitative approaches for contextualising suboptimal sleep outcomes and support the need for interventions that address upstream influences on sleep health.

## Supplementary Information

Below is the link to the electronic supplementary material.

Supplementary Material 1

## Data Availability

The interview manuscripts that support the findings of this study are available on Open Science Framework: https://doi.org/10.17605/OSF.IO/H3F6B.
