# Supplementary material for: Sleeping but struggling: a qualitative study of the lived experiences of sleep in student-athletes
Source: Sci Rep. 2026 Jun 2;16:25038. doi: 10.1038/s41598-026-55657-9 (PMC13462718; doi:10.1038/s41598-026-55657-9)
Supplement: Supplementary file 1 [file 41598_2026_55657_MOESM1_ESM.docx]

**Supplementary Material**

**Interview guide**

**Section one**

*** Note all topics mentioned during this section***

To start, can you discuss with me about your general sleep as a student-athlete?

*Prompt: What things related to being a student-athlete do you think are (a) beneficial/(b) challenges to your sleep?*

*Prompt: What additional benefits or challenges do you face that (a) student peers/(b) elite athletes may not?*

*Prompt: Are you generally satisfied or dissatisfied with your sleep? Why is this?*

**Section two**

*** Note all topics mentioned during this section***

Provide athlete with visual sleep report. Provide an overview and mention that the results will be discussed in more detail after the interview.

Looking at the sleep summary, can you reflect on some of the days when you managed (a) more sleep/(b) less sleep?

*Adjust if there are specific days of interest noted during the sleep report*

*Prompt:* *Can you comment on your sleep beyond the details presented in the sleep report (e.g., napping)?*

**Section three**

Next, we are going to discuss some of the things mentioned as being beneficial to sleep earlier.

1. You mentioned “Topic A”, can you tell me more about this
2. Can you give me an example of this?
3. Tell me more about this example

*Repeat for each topic listed*

Now, we are going to discuss some of the things mentioned as being challenges to sleep earlier.

1. You mentioned “Barrier A”, can you tell me more about this
2. Can you give me an example of this?
3. Tell me more about this example
4. Reflecting on “Barrier A”, do you have any suggestions as to how this challenge could be addressed?

*Repeat for each barrier listed*

Do you have any further topics around sleep that you believe are important considerations but have not previously been discussed?
